# Impact of Social Isolation Due to COVID-19 on VA Home-Based Primary Care Veterans and Caregivers

**DOI:** 10.1093/geroni/igab046.2208

**Published:** 2021-12-17

**Authors:** Chelsea Manheim, Nelly Solorzano, Juli Barnard, Tamar Wyte-Lake, Leah Haverhals

**Affiliations:** 1 Denver Center of Innovation for Veteran Centered and Value Driven Care (COIN), Denver, Colorado, United States; 2 Department of Veterans Affairs, Aurora, Colorado, United States; 3 US Department of Veterans Affairs, U.S. Department of Veterans Affairs (VA), California, United States

## Abstract

In December 2020 we began conducting phone interviews with Veterans, and their caregivers, receiving care through the United Sates (US) Department of Veterans Affairs (VA) Home Based Primary Care (HBPC) program. Our goal was to describe experiences of Veterans and caregivers managing changes in care delivery related to the COVID-19 pandemic and navigating increased social isolation due to social distancing. We interviewed 38 Veterans (average age 78) and caregivers (average age 62) across seven VA HBPC programs. Findings showed those living in their own homes found increased isolation more manageable than those living in assisted living facilities, which restricted visitors. Caregivers had a harder time managing isolation than Veterans, as Veterans were used to being primarily homebound. Veterans and caregivers relied on increased phone communication with their HBPC teams, with some began participating in virtual visits. Implications include insights into better supporting older, homebound adults and their caregivers during disasters.

